# Apela promotes blood vessel regeneration and remodeling in zebrafish

**DOI:** 10.1038/s41598-023-50677-1

**Published:** 2024-02-14

**Authors:** Nicolas Nys, Abdel-Majid Khatib, Geraldine Siegfried

**Affiliations:** 1 RYTME, Bordeaux Institute of Oncology (BRIC)-UMR1312 Inserm, University of Bordeaux, B2 Ouest, Allée Geoffroy St Hilaire CS50023, 33615 Pessac, France; 2 ZebraFish, Research and Technology, B2 Ouest, Allée Geoffroy St Hilaire CS50023, 33615 Pessac, France; 3 Bergonié Institute, Bordeaux, France

**Keywords:** Peptides, Cell biology

## Abstract

In contrast to adult mammals, zebrafish display a high capacity to heal injuries and repair damage to various organs. One of the earliest responses to injury in adult zebrafish is revascularization, followed by tissue morphogenesis. Tissue vascularization entails the formation of a blood vessel plexus that remodels into arteries and veins. The mechanisms that coordinate these processes during vessel regeneration are poorly understood. Hence, investigating and identifying the factors that promote revascularization and vessel remodeling have great therapeutic potential. Here, we revealed that fin vessel remodeling critically depends on Apela peptide. We found that Apela selectively accumulated in newly formed zebrafish fin tissue and vessels. The temporal expression of Apela, Apln, and their receptor Aplnr is different during the regenerative process. While morpholino-mediated knockdown of Apela (Mo-Apela) prevented vessel remodeling, exogenous Apela peptide mediated plexus repression and the development of arteries in regenerated fins. In contrast, Apela enhanced subintestinal venous plexus formation (SIVP). The use of sunitinib completely inhibited vascular plexus formation in zebrafish, which was not prevented by exogenous application. Furthermore, Apela regulates the expression of vessel remolding-related genes including VWF, IGFPB3, ESM1, VEGFR2, Apln, and Aplnr, thereby linking Apela to the vascular plexus factor network as generated by the STRING online database. Together, our findings reveal a new role for Apela in vessel regeneration and remodeling in fin zebrafish and provide a framework for further understanding the cellular and molecular mechanisms involved in vessel regeneration.

## Introduction

The development of new blood vessels is a critical process during embryogenesis and growth^1,2^, as well as in regenerative processes such as tissue renewal and wound healing^3^. New blood vessel formation involves restricted synchronization of different cellular processes, such as proliferation and migration. During the early embryonic stages, vascular development habitually starts with the establishment of vascular networks with comparable shapes and assemblies^4^. In contrast, at later stages, some vascular structures appear through vessel plexus formation, which in turn remodels into a well-organized network of veins and arteries, as observed during vessels of regenerating tissues, such as following zebrafish fin amputation^5,6^ or during blood vessel formation in the mouse retina^7^.

Angiogenesis, the development of new blood vessels from existing vessels, is regulated by various proangiogenic factors such as VEGF^8,9^. These mediators can be produced and secreted by various cells, including endothelial, stromal, and immune cells, and mediate the vessel sprouting required for vessel formation and remodeling^8^. Indeed, angiogenic molecules promote initial plexus formation, followed by progressive blood vessel differentiation, leading to organized artery and vein networks^5,10^. Therefore, while angiogenesis seems to be required for recovery from tissue injury^1,2^, abnormal angiogenesis can also contribute to the emergence and progression of various pathological conditions, including cancer and neurodegenerative and cardiovascular diseases^11,12^.

Recent studies have indicated the importance of apela in various physiological and pathological processes^13^. Apela is a peptide with 55‐amino acid secretory precursors. Following cleavage by the proprotein convertase Furin, the Apela precursor generates a 32‐amino acid mature peptide (Apela‐32)^14,15^. Although reduced homology levels were found between Apela and Apln peptides, in vitro studies revealed that Apela can bind to Apln receptors (Aplnr) in zebrafish and mediate receptor internalization and activation^16,17^. Zebrafish embryos with deficient Apela or Aplnr genes show comparable cardiac development malformations, indicating a key role of Apela in embryonic cardiovascular development^16,17^. Further studies have revealed that the temporal expression of Apela and Apln are different during zebrafish embryo development and seem to act during the early migratory process of angioblast migration and heart development^16–18^. In mice, loss of apela also promotes cardiovascular malformations and embryonic lethality in less than 50% of cases^19,20^. In vitro studies revealed that Apela‐32 can promote the formation of vascular tubular structures^15,21,22^. To date, the role of Apela in vessel formation and remodeling during fin regeneration in zebrafish remains to be elucidated. In the current study, we found that Apela plays a crucial role in regenerative angiogenesis.

## Results

### Apela is upregulated in the blastema during zebrafish caudal fin regeneration

**Figure 1:**
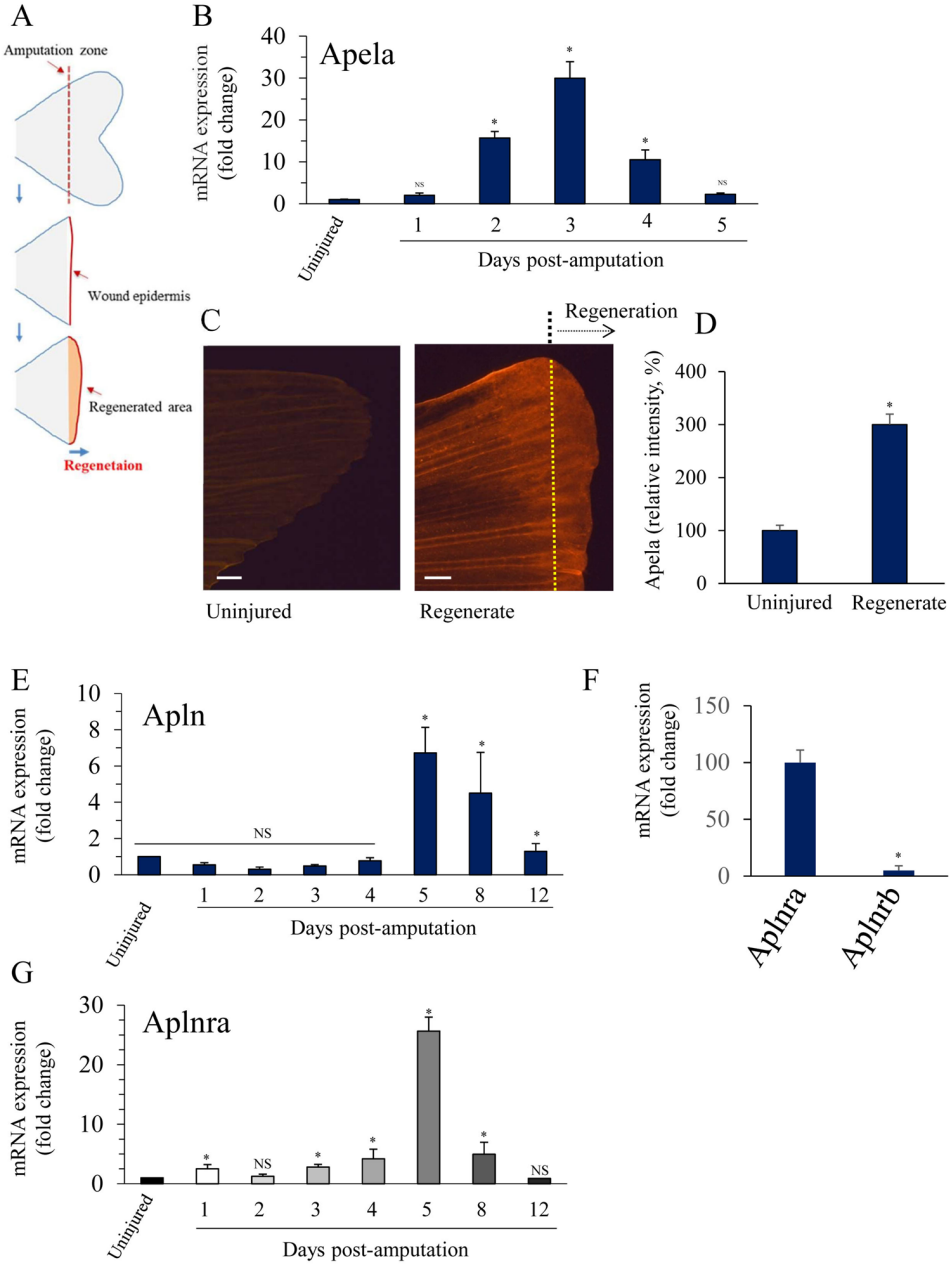
Apela is upregulated during zebrafish caudal fin regeneration. (**A**) Schematic representation of experimental plan showing amputation site and regenerated fin areas. Adult zebrafish were anesthetized in tricaine and fins were amputated and allowed to regenerate for indicated time periods. (**B**) Total RNA was isolated from fins (15–20 fins per time point) with uncut fins as controls (0 dpa) and analyzed by real-time PCR using specific primers for Apela or β-actin. Results are shown in the bar graph and are expressed as the ratio of the indicated transcripts relative to control (0 dpa). Results are shown as means ± S.E. of three experiments performed in triplicate. (**C**) Representative confocal images of control fins (0 dpa) and at 3 dpa (*n* = 12) subjected to immunofluorescence of Apela (red signal). (**D**) Quantification of fluorescence intensity in ImageJ. (**E**) Total RNA was isolated from fins (15–20 fins per time point) with uncut fins as controls (0 dpa) and analyzed by real-time PCR as in (**A**), using specific primers for Apln. (**F**) Aplnra and Aplnrb expression analysis by RT-PCR in uncut fins. (**G**) Aplnra expression analysis as in (**A**). The mean ± S.E values are shown. *p < 0.05. Scale bars represent 0.5 mm.

Dramatic changes in zebrafish vessel morphology occur during caudal fin vessel formation and remodeling^23^, suggesting that some regulated processes of angiogenesis are involved in zebrafish fin vessel regeneration. The importance of Apela and its receptor Aplnr in various physiological and pathological angiogenesis processes is now well established^15–17,19^, however, little is known about its role in vessel regeneration and remodeling. To assess whether these processes involve Apela, we first amputated the caudal fins of adult zebrafish and analyzed the expression of Apela 5 days post amputation (dpa) using real-time PCR. As observed in Fig. 1A, only low constitutive levels of Apela were detected in fins obtained from controls and non-amputated zebrafish fins. Following amputation, Apela was markedly upregulated in the fin, proximal to the injury area, and in the blastema at 2–4 dpa, reaching maximal levels at 3 dpa, then gradually decreasing to nearly normal levels during the rest of the fin regeneration (Fig. 1A,B). To explore the expression of Apela at the protein level during fin regeneration, we used a specific Apela antibody and immunohistochemical analysis. As illustrated in Fig. 1C, induced expression of Apela was observed in regenerated fins at 3 dpa, compared to controls (Fig. 1D). High levels of Apela were detected mainly in the periphery of the regenerating area. Interestingly, compared with uninjured fins, enhanced expression of Apela was also observed upstream of the amputated area (Fig. 1B–D). Similarly, using specific primers for Apln and the two Apela receptors (Aplnra and Aplnrb), real-PCR analysis of regenerated fins at indicated dpa periods revealed that following fin amputation, zebrafish Apln (Fig. 1E) was also markedly upregulated in the regenerated area, reaching maximal levels at 5–8 dpa, and then progressively decreasing to almost normal levels. Analysis of Aplnra and Aplnrb expression in zebrafish revealed that Aplnra was the most expressed receptor in zebrafish fins and that Aplnrb was weakly expressed (Fig. 1F). Therefore, we analyzed the expression of Aplnra during fin regeneration. As illustrated in Fig. 1G, Aplnra expression was markedly upregulated during fin regeneration, with maximal levels at 5 dpa, which decreased to normal levels thereafter. Taken together, these data suggest that the significant upregulation of Apela in regenerating blastema tissue may be implicated in the progressive replacement of the amputated structures of the fin.

### Blockade of Apela expression suppresses vessel differentiation

**Figure 2:**
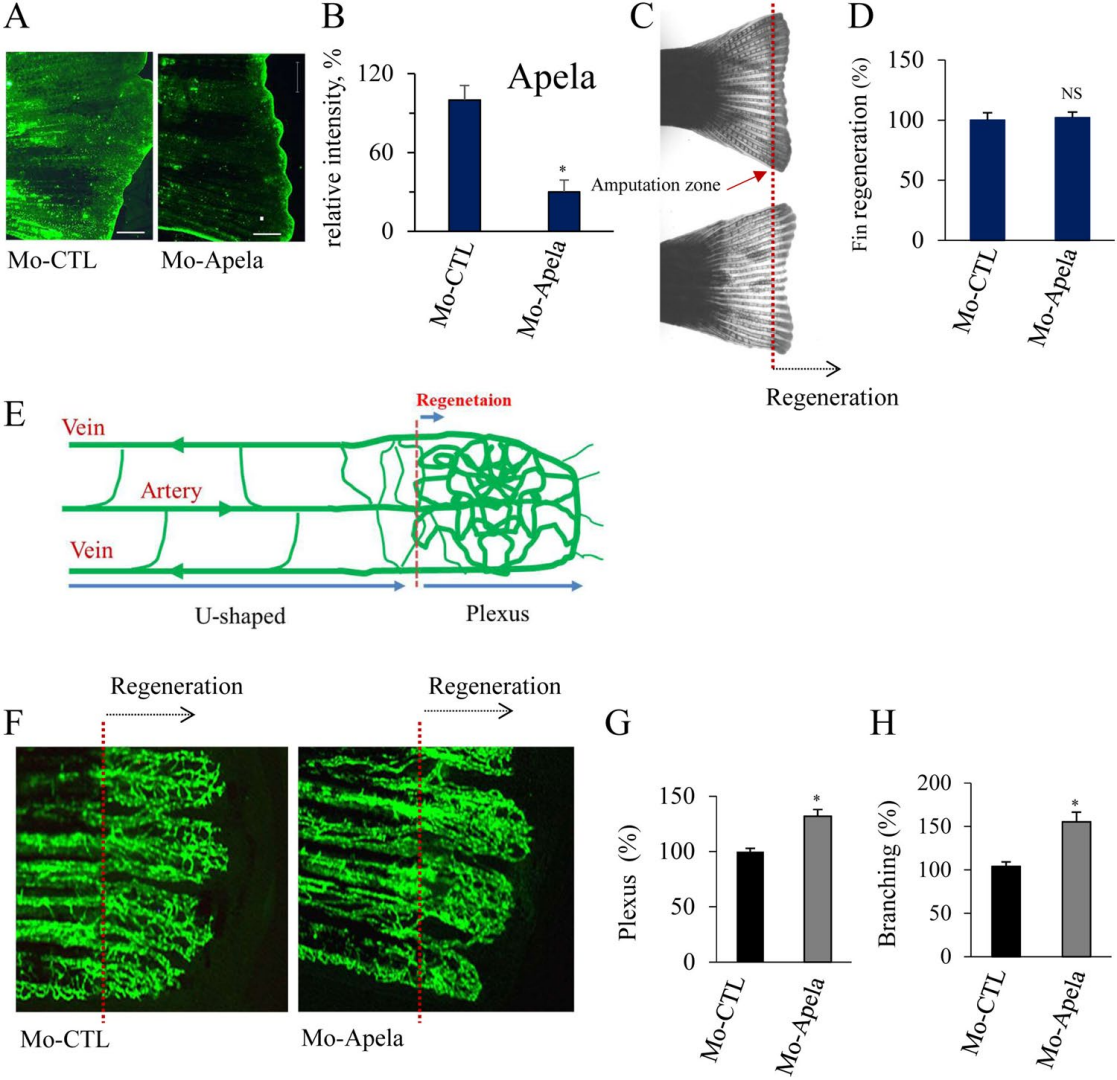
Blockade of Apela expression suppresses vessel differentiation. (**A**, **B**) Representative confocal images of caudal fins of adult zebrafish (15–20 fins/per experiment) that were electroporated with control Mo-CTL and Mo directed against Apela (Mo-Apela), and fin were allowed to regenerate for 3 days prior Apela expression analysis by immunohistochemistry using an anti-Apela. (**C**, **D**) MO-Apela had no effect on total fin regeneration. (**E**) Schematic representation of anastomotic bridge with emerging sprouts forming vascular plexus and the U-shaped formed by the artery and the two veins. (**F**) Inhibition of Apela by MO-Apela prevents remodeling of immature vascular network during fin regenerating in *Tg(fli:EGFP)*^*y*^, as observed with the enhanced plexus (**G**) and branching (**H**) vasculature. Results are shown as means ± S.E. of three experiments performed in triplicate. *p < 0.05. NS, not significant.

To evaluate the importance of Apela in fin regeneration and vessel differentiation, we assessed the effect of antisense morpholino oligo (Mo) knockdown on zebrafish caudal fin regeneration. This specific inhibitor of translation acts by binding to complementary sequences on mRNA and inhibiting ribosome access^24^. Caudal fins of adult zebrafish were electroporated with control Mo-CTL and Mo directed against Apela (Mo-Apela), and the distal part of the fin was sectioned and allowed to regenerate for three days prior to Apela expression analysis by immunohistochemistry. As illustrated in Fig. 2A,B, the expression of Mo-Apela in regenerated fins inhibited Apela expression compared to controls. However, no significant effect was observed on total fin regeneration (Fig. 2C,D).

After fin amputation, an anastomotic bridge was formed with emerging sprouts, leading to the formation of a vascular plexus (Fig. 2E,F). These newly formed branches were smaller and had abundant connections with their adjacent vessels. At this step, there are no noticeable morphological differences between arteries and veins in the formed blood vessel plexuses^5,6,12,25^. During plexus formation, redeveloping vessels are progressively remodeled to form arteries and veins at further proximal points, as evidenced by a thickened central vessel that extends from the artery in the stump (U-shaped)^5,6,12,25^. Formation of this plexus mainly occurs between 3 and 8 dpa^25^. Therefore, we investigated the effect of Mo-Apela repression on vascular plexus formation and vessel regeneration. To this end, we used a transgenic zebrafish Tg (fli1: EGFP) line that expresses EGFP in all endothelial cells under the control of the zebrafish fli1 promoter^6,26^. In a normal regenerated fin, plexus formation is more limited with a well-defined U-shaped vascular structure with a thickened central vessel with more intense EGFP fluorescence, which extends from the artery in the stump (Fig. 2F).

Although total fin regeneration was not significantly affected in caudal fins electroporated with Apela-MO (Fig. 2C,D), the latter significantly inhibited fin vessel remodeling and the U-shaped connection process (Fig. 2E–H). As illustrated, blood vessels appeared denser with the continuing formation of vessels at the distal tips of the regenerating fins, indicating intense vessel formation and reduced vessel remodeling. There is no clear morphological distinction between arteries and veins in the regenerating blood vessel plexuses, suggesting a continuity of blood vessel regeneration (Fig. 2F–H)^27^. Thus, Apela induction appears to participate in the remodeling of the immature vascular network during fin regeneration.

### Exogenous Apela peptide mediates fin vessel differentiation during regeneration

**Figure 3:**
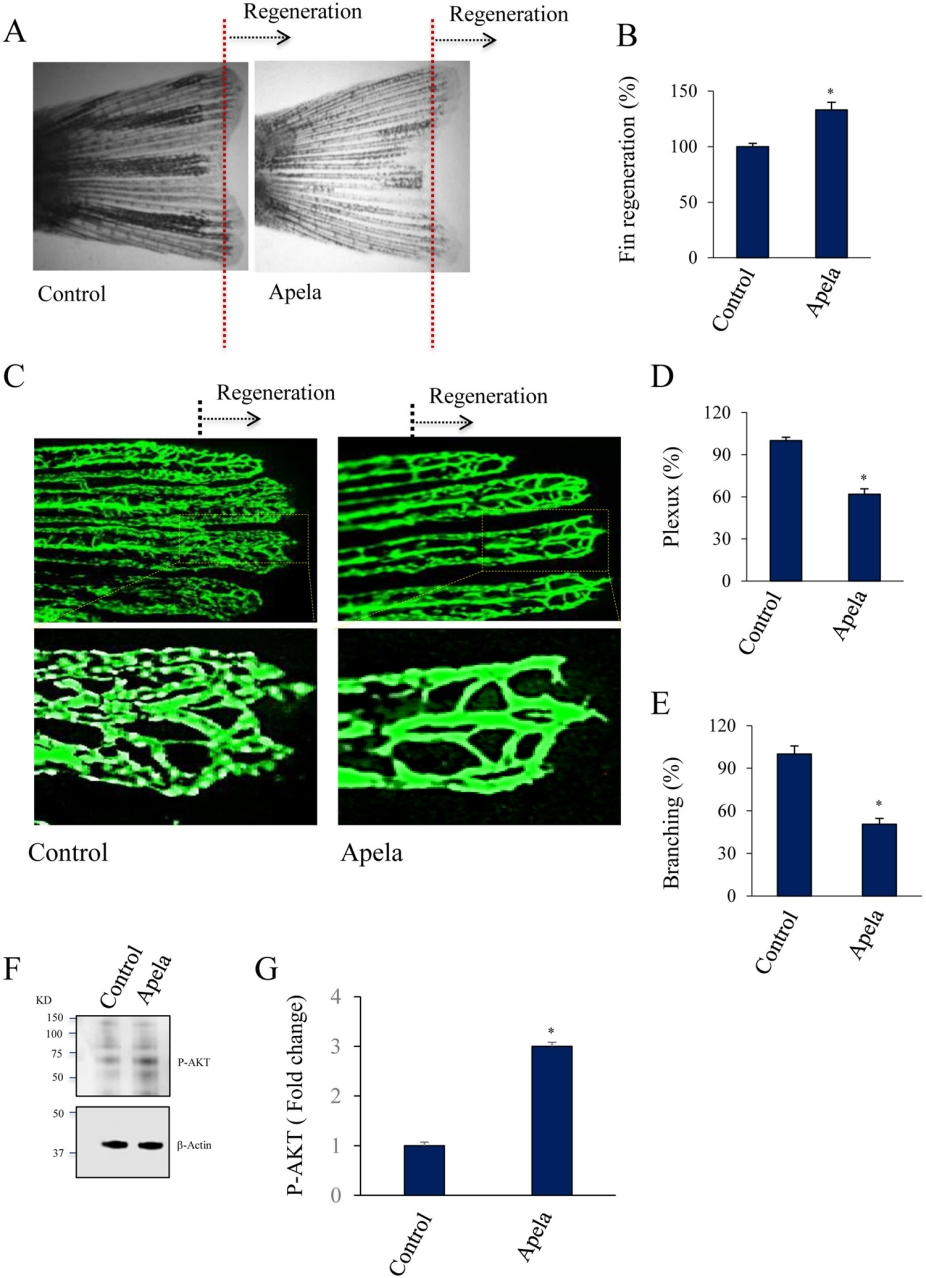
Apela mediates fin vessel differentiation during regeneration. (**A**, **B**) *Tg(fli:EGFP)*^*y1*^ zebrafish exposed to Apela (10 μM) after fin amputation and were allowed to regenerate prior analysis. (**C**, **E**) *Tg(fli:EGFP)*^*y1*^ fins (20 fins/per experiment) treatment with Apela (10 μM) shows reduced vasculature plexus (**D**) and branching (**E**). (**F**, **G**) Western blot analysis of the activation of AKT in control fins and after 15 min treatment with Apela (10 μM). Images shown in upper and lower panels were cropped from the same blot. Full blots are provided in Supplementary Fig. 1. All results shown are representative of at least 3 independent experiments with means ± S.E and *p < 0.05.

To provide additional evidence regarding the role of Apela in vessel regeneration and differentiation, zebrafish caudal fins were amputated at the midfin level, and the fish were immediately placed in either water alone or containing Apela (10 μM) to allow fin regeneration for 3 days. As illustrated in Fig. 3A,B, Apela enhanced fin regeneration compared with the controls. Analysis of blood vessels in the regenerated fins of *Tg(fli1:EGFP)* fish revealed that exposure to Apela reduced plexus formation (Fig. 3C,D) and vessel branching (Fig. 3E) and induced the formation of a U-shaped structure, as evidenced by a thickened central artery (Fig. 3C). Binding of Apela to its receptor Aplnr activates the AKT signaling pathway, which is required for vessel remodeling and structure^15,21,22^. To test whether Apela can also activate AKT during fin regeneration, we directly evaluated AKT phosphorylation by western blotting in regenerated fins incubated with Apela (10 μM). Zebrafish caudal fins were amputated at the midfin level and allowed to recover in the absence and presence of apela. AKT phosphorylation analysis at 3 dpa revealed that Apela induced significant AKT activation by up to 3-fold (Fig. 3F,G).

### Sunitinib and Apela cross talk during vascular plexus regeneration and remodeling

Previously, the antiangiogenic effect of sunitinib, a small-molecule receptor tyrosine kinase inhibitor, was thought to be mediated by the inhibition of cellular signaling of multiple angiogenic targets, such as PDGFR and VEGFR^28^.

**Figure 4:**
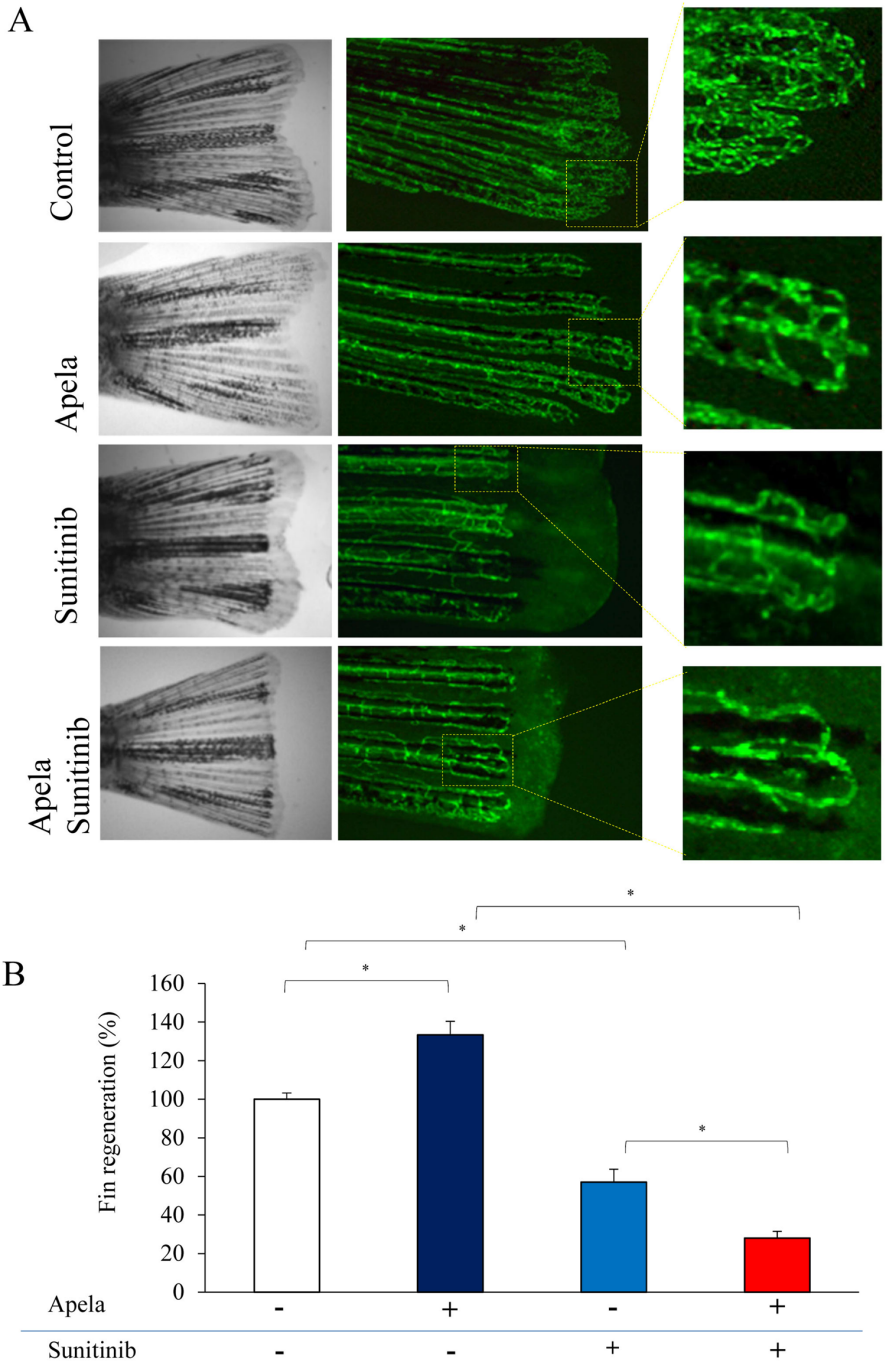
Sunitinib and Apela cross talk during vascular plexus regeneration and remodeling. (**A**) Zebrafish (15–20/per experiment) exposed to Sunitinib (0.5 μM) in the absence and presence of Apela (10 μM) after fin amputation and allowed to regenerate during 3 days prior analysis. (**B**) Results are shown as means ± S.E. of three experiments performed in triplicate. *p < 0.05.

To evaluate whether Apela activity regulates endothelial cell sensitivity to sunitinib, as previously observed for other angiogenic factors such as FGF^29^, we evaluated the effect of sunitinib on vascular plexus regeneration and remodeling during fin regeneration. Following *Tg(fli1:EGFP)* fish caudal fin amputation, Apela induced the formation of the U-shaped structure regenerated vessels with more directional plexus growth and less interbranching, that correlate with increased regeneration. This process was completely blocked by sunitinib (Fig. 4).

### Apela and vascular plexus remodeling molecule network

**Figure 5:**
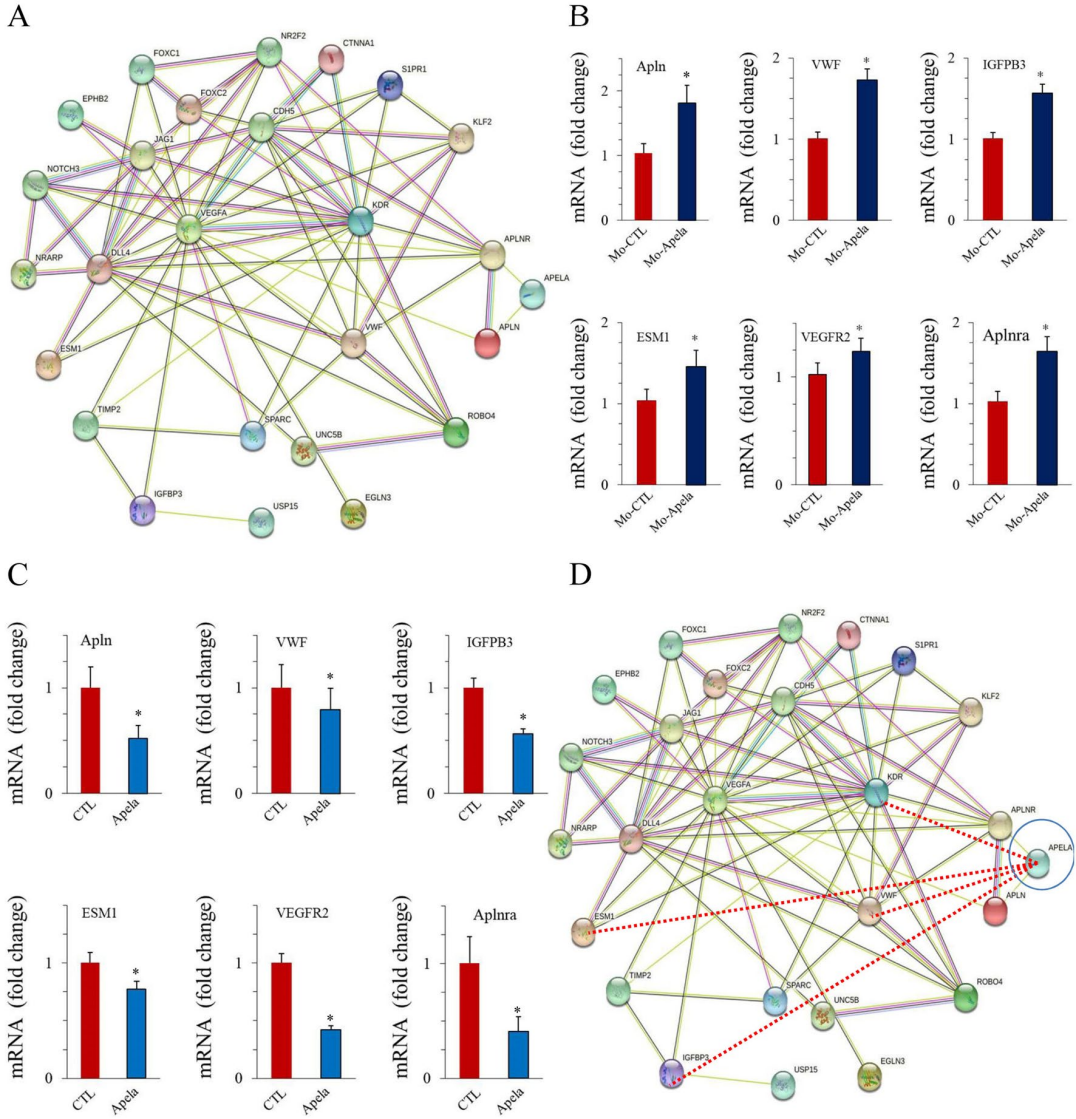
Apela and vascular plexus remodeling molecule network. (**A**) The STRING network view. Combined screenshots from the STRING website, which has been queried with a subset of proteins involved in the formation and remodeling of vascular vessels (Notch, VEGF, Ephrin, Hedgehog, TGF, COUP-TFII, FOXC2 and others. Colored lines between the proteins indicate the interaction evidence. (**B**, **C**) Relative expression of indicated VWF, IGFPB3, ESM1, VEGFR2, Apela, Apln and Aplnr mRNA in pooled fin zebrafish (15–20/per experiment) treated with Apela (**B**) or Mo-Apela (**C**). (**D**) STRING network queried with the same subset of proteins in (**A**) and the set of the angiogenic proteins: VWF, IGFPB3, ESM1, VEGFR2, Apela, Apln and Aplnr. Red lines between the proteins indicate the new interaction evidence. For (**B**) and (**C**), results are shown as means ± S.E. of three experiments performed in triplicate. *p < 0.05.

During blood vessel formation, the interaction of various cells, such as endothelial cells (ECs), pericytes, and macrophages, is required to enable effective vessel formation and remodeling. These interactions are promoted by various molecules network regulating different functions ranging from endothelial sprouting, vascular plexus formation and remodeling and stabilization, such as Notch, VEGF, Ephrin, Hedgehog^4^, TGF, COUP-TFII, FOXC2 and others^30,31^ (Fig. 5A). To evaluate the potential implications of Apela in this network, we first used the STRING online database (https://string-db.org/) to search for functional protein association networks among Apela, Apln, Aplnr, and these genes. Although interactions between Apln, Aplnr, and these angiogenic genes were observed, no direct implication of Apela in this vascular plexus network was documented (Fig. 5A). To investigate the potential interaction between Apela and the main genes involved in vascular plexus remodeling, we analyzed the effect of Apela repression by Mo-Apela on the expression of several genes with key roles in vessel remodeling in regenerated fins after 3 dpa, including VWF, IGFPB3, ESM1, and VEGFR2. We also evaluated the effect of apela on the expression of Apln and Aplnr. As illustrated in Fig. 5B, we found that Apela repression by MO-Apela enhanced the expression of all the analyzed genes. Accordingly, incubation of regenerated fins with Apela peptide reduced the expression of all the analyzed genes (Fig. 5C).

Interestingly, the ability of Apela to inhibit Aplnra expression was also observed during fin regeneration (Fig. 1G), which may explain the temporal disconnection between the expression of Apela and aplnra. These findings uncover the role of upstream regulatory genes in vessel remodeling. We then built a new schematic diagram based on the network linking Apela to VWF, IGFBP3, ESM1, VEGFR, Apln, and Aplnr genes that showed the novel Apln signaling links to the keystone angiogenesis genes and vessel remodeling (Fig. 5D).

### Effect of Apela on sub-intestinal venous plexus (SIVP) formation

**Figure 6:**
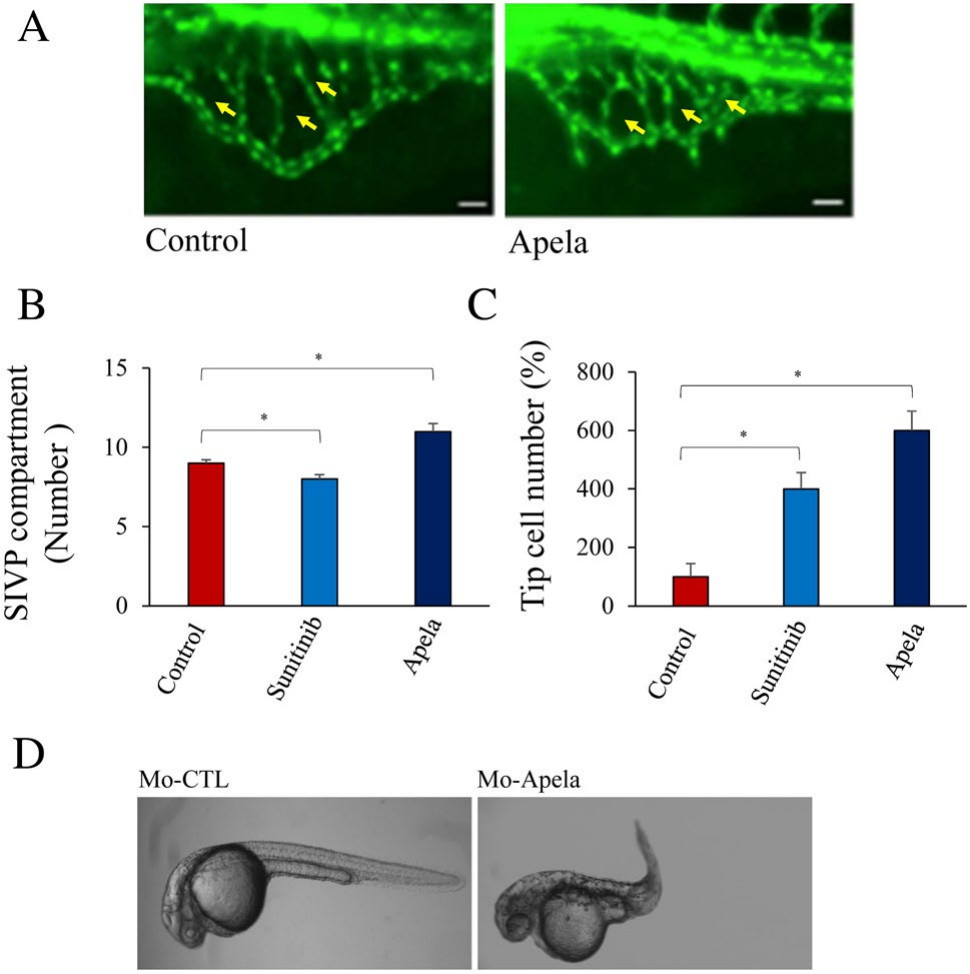
Effect of Apela on sub-intestinal venous plexus (SIVP) formation. (**A**–**C**) *Tg(fli:EGFP)*^*y1*^ embryos zebrafish (15–20/per experiment) were treated with Apela and sunitinib (for comparison) and the number of SIV compartments (**B**) and tip cells (**C**) were enumerated. Yellow arrowheads point to compartments. (**D**) Injection of MO-Apela into zebrafish embryos at the 1–4 cell stage, significantly affected normal embryonic development. For (**B**) and (**C**), results are shown as means ± S.E. of three experiments performed in triplicate. *p < 0.05.

Previously, the vessels of the sub-intestinal venous plexus (SIVP) were reported to be insensitive to several angiogenic factors and alterations in their pathways, such as the blockade of Shh, Notch, and Pdgf signaling^4,32^. Therefore, we assessed whether Apela mediated comparable angiogenic effects in SIVP and fin vessels. Therefore, we examined SIVP formation in *Tg(fli:EGFP)*^*y1*^ embryos in the absence and presence of the Apela peptide at 3 dpf. At 3 dpf, the SIVP appeared as a vascular basket with compartments delimited by the veins (Fig. 6A). In the presence of Apela, we observed an increased number of SIV compartments, greater expansion of the yolk, and a higher number of sprouts from the basket (Fig. 6A,B). We also detected enhanced tip cell numbers in the presence of Apela (Fig. 6A,C). This finding suggests that the effect of Apela on vessel formation and remodeling differs during fin regeneration and SIVP formation. Similarly, we directly assessed the effect of Apela repression on embryonic development, which requires angiogenesis and vessel remodelling. We found that injection of MO-Apela into zebrafish embryos at the 1–4 cell stage significantly affected normal embryonic formation (Fig. 6D).

## Discussion

Induction or inhibition of functional blood vessel formation in various pathologies such as ischemic vascular disease, age-related macular degeneration, or cancer based on the modulation of angiogenesis showed important clinical advantages; however, a single pathway targeting anti-angiogenesis therapies in cancer faces the problem of resistance or alternative modes of vessel growth, and combinatorial targeting seems to be the best approach^33,34^; however, in most cases, tumors can continue to grow, or even become more metastatic in the absence of angiogenesis^35^.

The most successful and widespread clinical use of anti-angiogenic agents has been in the treatment of ocular diseases^36,37^. Vascular normalization for effective chemotherapeutic delivery is another use of anti-angiogenesis agents, but so far, with relatively limited use^38,39^. It is clear that blocking angiogenesis is much easier than promoting it. Indeed, waking up dormant vasculature and inducing effective and functional vascular growth is far more challenging, but also has great potential clinical benefit. Simply promoting endothelial cell proliferation may not be the best way to achieve effective tissue vascularization and healing. Therefore, we must understand and mimic the mechanisms used by developing tissues as much as possible. Achieving a proper spatiotemporal mitogenic and sprouting balance with pharmacological compounds is key. First, we need to understand what regulates the bell-shaped response of endothelial cells to mitogenic stimulation, and find ways to achieve a therapeutic equilibrium to achieve maximal sprouting and proliferation. In this study, we evaluated the involvement of Apela expression in vessel formation and remodeling in a zebrafish fin vessel regeneration model. Apela is expressed by endothelial stem cells and during embryonic development^40,41^. Our results showed that Apela expression is significantly induced in zebrafish fins during the early stages of fin regeneration, compared to Apln and Aplnr expression. Vascular plexus differentiation was significantly increased in the presence of Apela, and repression of Apela expression by Mo-Apela maintained the undifferentiated vascular plexus. Moreover, Apela induced phosphorylation of Akt in regenerating fins, suggesting that the Akt pathway might be involved in Apela-induced vascular plexus differentiation and fin regeneration. These results provide preliminary evidence that apela is involved in vascular plexus differentiation during fin regeneration. Our study also showed that sunitinib abolished fin vessel regeneration and that Apela did not reverse sunitinib-induced vessel repression, indicating that Apela may not interfere with the anti-angiogenic effect of sunitinib, as previously reported for other factors such as FGF2^29^. Previously, sunitinib, which suppresses the migration and proliferation of endothelial cells and the new formation of intersegmental vasculature in zebrafish, was associated with eliminated fenestration-like holes on the endothelium and induced thicker vascular walls, suggesting that sunitinib induces vascular maturation independent of angiogenic factors, while deteriorating newly formed vasculature^42^. Therefore, in our model, the inability of Apela to rescue vessel formation and remodeling in sunitinib-treated zebrafish fins may be linked to the absence of new vessels modulated by Apela.

It is clear that for angiogenesis and vascular plexus remodeling to proceed effectively during physiological and pathological conditions, it is essential that a complex array of angiogenic and anti-angiogenic factors, interacting with multiple cells and tissues, is tightly regulated^43^. The ultimate therapeutic goals, which are to mitigate angiogenesis during pathological processes such as inflammation and tumorigenesis and to enhance angiogenesis to prevent and/or treat ischemic events, have become realizable at the clinical level, and thus capture the imagination and attention of all those involved in health care and research^44^. During vascular plexus formation and arterial-venous specification, crosstalk between various signaling pathways implicated in early endothelial cell development plays a significant role in vessel remodeling and arterial-venous specification. The major crosstalk, the activation of Notch signalling following VEGF-A, binds to VEGFR2^45^. During this pathway, the activation of arterial-specific genes, including EphrinB2, is upregulated downstream, whereas venous-specific EphB4 expression is suppressed^46^. Similarly, β-catenin, a transcriptional co-activator of the Wnt signalling pathway, upregulates the Notch ligand Dll4 and promotes arterial specification^47^. In addition, hedgehog acts upstream of VEGF-A via a smoothened receptor to drive arterial endothelial cell specification and repress venous fate^48^. In addition, the COUP-TFII transcription factor was found to regulate these processes by suppressing Notch signalling^49^. The propagation of lymphatic endothelial cells is mediated by VEGFR3 signaling, driven by VEGF-C from the surrounding mesenchyme, and stabilization and quiescence of the lymphatic system are mediated by FOXC2^50^. Similarly, Apln and Aplnr functions are also required for vascular outgrowth and alignment of arteries and veins^51^. Indeed, a recent study revealed that zebrafish lacking apelin signaling exhibit defects in endothelial tip cell morphology and sprouting^52^. VWF can also interact with αvβ3 and induce arterial maturation during vascular development^53^. IGFBP-3 can serve as a modulator of vascular development by potently regulating bone marrow-derived cell (BMDC) function, specifically by stimulating endothelial precursors to differentiate into endothelial cells and promoting their migration^54,55^. Esm1 modulates Endothelial Tip Cell Behavior and Vascular Permeability by Enhancing VEGF Bioavailability^56^. Using the STRING online database, the majority of these genes were identified to show established crosstalk, and real-time PCR analysis revealed the involvement of Apela in the regulation of several genes involved in vessel remodeling, thereby linking Apela to this angiogenic network.

Analysis of the effect of Apela on the sub-intestinal venous plexus (SIVP) revealed that Apela enhanced SIVP formation, highlighting differences in the sensitivity of the SIVP and fin vessel plexus to Apela. Previously, a similar observation was made for several angiogenic factors and/or their inhibition. SIVP was reported to be insensitive to Notch, SHH, and PDGF inhibition but showed high sensitivity to VEGF and BMP inhibition in other vessel beds. In the current study, we propose that Apela participates in an angiogenic network that operates as a vessel-remodeling factor during fin vessel regeneration in zebrafish, which is not the case during sub-intestinal venous plexus formation.

## Methods

### Zebrafish lines and fin regeneration assay

Wild-type zebrafish (*Danio rerio*) and transgenic zebrafish *Fli*-EGFP-Tg were purchased from the ZIRC fish center (Oregon) and housed under standard conditions in a core facility, and the water temperature was maintained at 28.5 °C^25^. For the zebrafish fin regeneration assay, adult fish of at least 10 weeks were anesthetized by the addition of 0.6 mM tricaine (ethyl-m-aminobenzoate, MS222, Sigma) to water. The caudal fins were amputated at a level proximal to the first bifurcation of the bony rays using razor blades. Animals were allowed to regenerate for various times at 28.5 °C depending on the experiment. Apela (Phoenix Pharmaceuticals) was injected intraperitoneally at 10 mg/ml in 10 ml. In another experiment, Apela was directly added to the zebrafish water (10 μM). For the experiments with the anti-angiogenic agent sunitinib (Sigma), 0.5 μM was also directly added in the fish water.

### Morpholino experiments

To block the activity of the Apela transcript, a synthetic morpholino antisense oligonucleotide (Mo-Apela) was designed and synthesized by GeneTools (Philomath, OR, USA), based on a previously reported sequence^57^, (https://zfin.org/ZDB-MRPHLNO-191209-4). The Mo Apela sequence was “TGGAAGAATCTCATGGTGATGCTCA.” As a control, Gene Tools standard in vivo control Mo “CCTCTTACCTCAGTTACAATTTATA.” was used in this study. To assess the effect of control Mo and Mo-Apela on fin regeneration, adult fish for at least 10 weeks were anesthetized by the addition of 0.6-μM tricaine to water. The caudal fins were amputated at a level proximal to the first bifurcation of the bony rays, and Mo and Mo-Apela (500 µM) were injected into the regenerates, as described previously^6^. In brief, following Mo and Mo-Apela injections, 10 consecutive 50-ms electric pulses at 15 V with a 1-s pause between pulses were applied via a pair of electrode disks (7 mm in diameter). Twenty-four hours post-injection, caudal fins were amputated at a level proximal to the first bifurcation with a scalpel, and fish were returned to a 28.5 °C tank. In other experiments, control Mo or Mo-Apela-Mo (500 µM) was injected into one-cell stage embryos.

### Real-time PCR

To determine the relative expression of Apela, Apln, and the two zebrafish Apela receptors (Aplnra and Aplnrb), VWF, IGFPB3, ESM1, and VEGFR2, zebrafish caudal fins were amputated and animals were allowed to regenerate for 3 days. Total RNA was isolated from 25 to 30 pooled fins and subjected to cDNA synthesis using the Superscript First Strand cDNA Synthesis System (Invitrogen). Relative quantification of specific mRNAs was performed by real-time PCR using the StepOnePlus™ Real-Time PCR System and Power SYBR Green PCR Master Mix (Applied Biosystems) according to the manufacturer's instructions^15,26^ using the following primers: (5-ACCAAACCACCCTGAGCATC-3ʹ) and (5-AGCGTTTCTTCGGGCAGTTG-3ʹ) for Apela, (5-TCTCCCTCCCATCCACACAC-3ʹ) and (5-TTGCTATGCTCGGTGGAGGC-3ʹ) for Apln, (5-CTGGGACTCACTGGGAATGG-3ʹ) and (5-GGGTCACCACAAAGGTCAGG-3ʹ) for Aplnra, (5-CAGGACAGGCTGTTGAGACA-3ʹ) and (5-TTGCAACATTTACGGGAACA-3ʹ) for VWF, (5-GACGCTTCCGCATTCCAAAC-3ʹ) and (5ʹ-CCTTTGGATGGACTGCACTGTT-3ʹ) for IGFPB3, (5ʹ-CGACTGTAAAGCTGGTGGACT-3ʹ) and (5ʹ-AACCCACTTCATTACCTGCTTCA-3ʹ) for ESM1, (5’-TCAACGTGGCTAAAGCGATAGA-3ʹ) and (5ʹ-TGAAGTGTATCTGAAGCATTG-3ʹ) for VEGFR2.

### The construction of the protein–protein interaction (PPI) network associated with Apela

We explored PPI using the Search Tool for the Retrieval of Interacting Genes/Proteins (STRING) database (http://stringdb.org/)^58^. Proteins involved in angiogenesis and vessel remodeling, as well as a list of the genes regulated by Apela, were used to interrogate the STRING database. The PPI relationships were analyzed using the STRING database with the required confidence (combined score) > 0.4 as the threshold. After the PPIs were examined, a PPI network was created using the STRING website. In the networks, the nodes correspond to proteins, and the edges represent interactions.

### Akt tyrosine phosphorylation

The caudal fins were amputated under anesthesia with tricaine and incubated with APELA (10 μM) for 15 min at 37 °C. Fins were washed twice in ice-cold PBS and lysed with 500 µl/dish in lysis buffer (50 mM HEPES (pH 7.6), 150 mM NaCl, 1% Triton X-100, 2 mM vanadate, 100 mM NaF, and 0.40 mg/ml phenylmethylsulfonyl fluoride)^15,26^. Equal amounts of protein were analyzed by western blotting for Akt phosphorylation using anti-phospho-Akt (Cell Signaling), as previously described^59,60^. Anti-Akt (Cell Signaling) was used for data normalization. The negative controls were directly incubated with the secondary antibody.

### Immunohistochemistry

For Apela detection, fin regenerates derived from transgenic zebrafish *Fli*-EGFP-Tg were incubated overnight at 4 °C with anti-Apela (Eurogentec). On the following day, the fins were washed and incubated with the secondary antibody. The negative controls were directly incubated with the secondary antibody. Photographs were obtained using a Zeiss Axioplan 2 Digital Imaging Microscope (Carl Zeiss Microscopy).

### Measurement

To measure the length of the regenerated fin and size of the fin plexus, only the third fin ray was used for comparison. The regenerated length was measured as the distance between the amputation level and the limit of the regenerated area (Fig. 1A). The size of the plexus was evaluated as the distance between the edge of the fin area with typical arteries and veins, and the end of the distal regenerated vessel plexus (Fig. 2E).

### Statistical analysis

All data are presented as mean ± standard error of the mean (SE) unless specifically mentioned. Data were analyzed using one-way ANOVA followed by Tukey’s multiple comparison test. All statistical analyses were performed using GraphPad Prism 6.0 (GraphPad software, La Jolla, CA, USA). p < 0.05 was considered to be statistically significant.

### Ethics statement

All methods were performed in accordance with relevant guidelines and regulations. Animal maintenance and procedures were approved by the Institutional Animal Care and Use Committee of the INSERM Institute and the University of Bordeaux in accordance with the ARRIVE guidelines.

### Supplementary Information

Supplementary Figure 1.

## Data Availability

All datasets and raw data are provided in this manuscript and are available to all the readers. Please contact the corresponding author for further information if necessary.
