# Supplementary material for: Apela promotes blood vessel regeneration and remodeling in zebrafish
Source: Sci Rep. 2024 Feb 14;14:3718. doi: 10.1038/s41598-023-50677-1 (PMC10867005; doi:10.1038/s41598-023-50677-1)
Supplement: Supplementary file 1 — Supplementary Figure 1. [file 41598_2023_50677_MOESM1_ESM.pdf]

Apela promotes blood vessel regeneration in zebrafish

Nicolas Nys, Abdel-Majid Khatib, Geraldine Siegfried

Full blots of Figure 3F, Upper panel

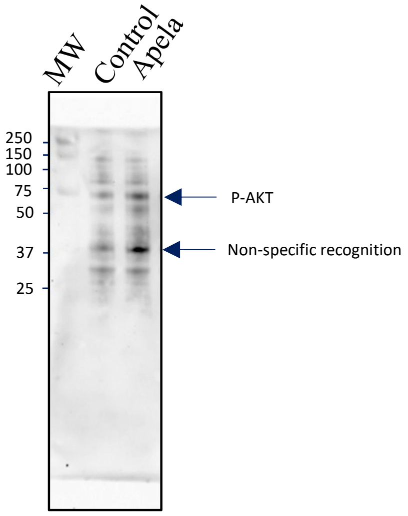

Full blots of Figure 3F, lower panel

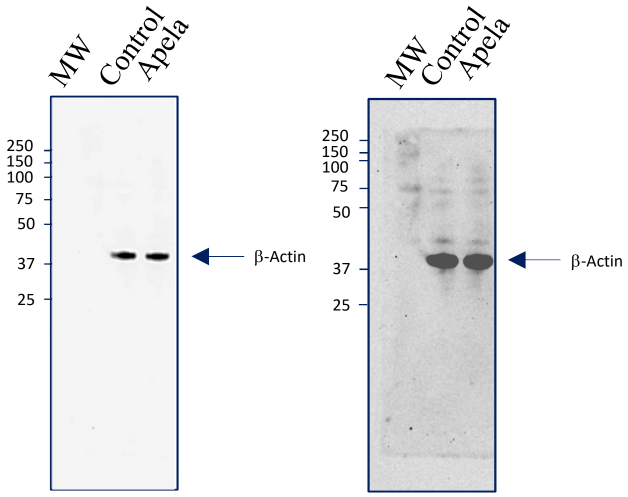

Same gel with high exposure and edge

Supplementary Fig. 1. Full blots of Figure 3F showing that the anti- P-AKT antibody exhibits a strong non-specific recognition of a protein lower than p-AKT. In the Figure 3F blots, whole cell lysates of zebrafish Fins were used on SDS-PAGE and P-AKT was detected using the anti-P-AKT antibody. Source data are provided as a Source Data file.
